# A cost analysis of intensified vs conventional multifactorial therapy in individuals with type 2 diabetes: a post hoc analysis of the Steno-2 study

**DOI:** 10.1007/s00125-018-4739-3

**Published:** 2018-10-06

**Authors:** Joachim Gæde, Jens Oellgaard, Rikke Ibsen, Peter Gæde, Emil Nørtoft, Hans-Henrik Parving, Jakob Kjellberg, Oluf Pedersen

**Affiliations:** 1 Novo Nordisk Foundation Center for Basic Metabolic Research, Section of Metabolic Genetics, Faculty of Health and Medical Sciences, University of Copenhagen, Blegdamsvej 3B, DK-2200 Copenhagen, Denmark; 2 Institute for Regional Health Research, University of Southern Denmark, Odense, Denmark; 3 Steno Diabetes Center Copenhagen, Gentofte, Denmark; 4 Department of Cardiology and Endocrinology, Slagelse Hospital, Slagelse, Denmark; 5 i2minds, Århus, Denmark; 6 Novo Nordisk A/S, Søborg, Denmark; 7 Department of Medical Endocrinology, Rigshospitalet, Copenhagen, Denmark; 8 VIVE, The Danish Center for Social Science Research, Herluf Trolles Gade 11, 1052 Copenhagen, Denmark

**Keywords:** Health economy, Multifactorial intervention, Organ complications, Type 2 diabetes

## Abstract

**Aims/hypothesis:**

Long-term follow-up of the Steno-2 study demonstrated that intensified multifactorial intervention increased median lifespan by 7.9 years and delayed incident cardiovascular disease by a median of 8.1 years compared with conventional multifactorial intervention during 21.2 years of follow-up. In this post hoc analysis of data from the Steno-2 study, we aimed to study the difference in direct medical costs associated with conventional vs intensified treatment.

**Methods:**

In 1993, 160 Danish individuals with type 2 diabetes and microalbuminuria were randomised to conventional or intensified multifactorial target-driven intervention for 7.8 years. Information on direct healthcare costs was retrieved from health registries, and the costs in the two groups of participants were compared by bootstrap *t* test analysis.

**Results:**

Over 21.2 years of follow-up, there was no difference in total direct medical costs between the intensified treatment group, €12,126,900, and the conventional treatment group, €11,181,700 (*p* = 0.48). The mean cost per person-year during 1996–2014 was significantly lower in the intensified treatment group (€8725 in the intensive group and €10,091 in the conventional group, *p* = 0.045). The main driver of this difference was reduced costs associated with inpatient admissions related to cardiovascular disease (*p* = 0.0024).

**Conclusions/interpretation:**

Over a follow-up period of 21.2 years, we found no difference in total costs and reduced cost per person-year associated with intensified multifactorial treatment for 7.8 years compared with conventional multifactorial treatment. Considering the substantial gain in life-years and health benefits achieved with intensified treatment, we conclude that intensified multifaceted intervention in high-risk individuals with type 2 diabetes seems to be highly feasible when balancing healthcare costs and treatment benefits in a Danish healthcare setting.

**Electronic supplementary material:**

The online version of this article (10.1007/s00125-018-4739-3) contains peer-reviewed but unedited supplementary material, which is available to authorised users.

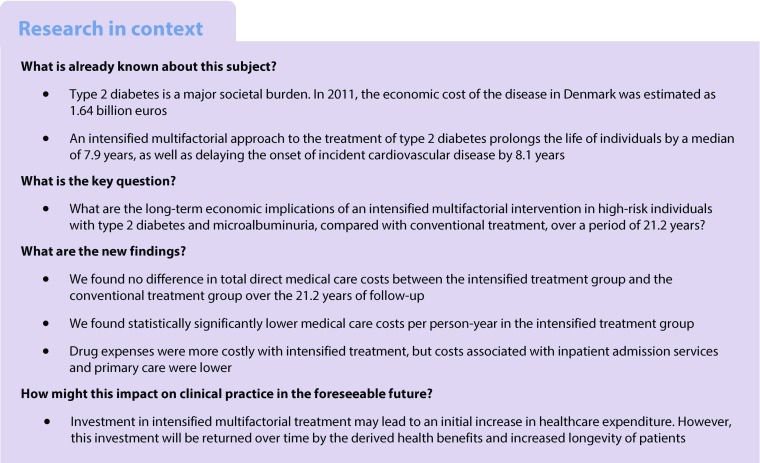



## Introduction

Cardiovascular disease (CVD) is the leading cause of death and disability among individuals with type 2 diabetes mellitus, with an incidence of almost three times that in the non-diabetic population [1]. The cardiovascular morbidity associated with type 2 diabetes poses a major health issue for individuals as well as an economic burden on the healthcare system. Individuals with type 2 diabetes have a life expectancy that is reduced by around 5 years and a disability-free life expectancy reduced by up to 9 years [2, 3].

Medical expenditure for Danish individuals with diabetes is, on average, higher than that for people without diabetes, ranging from 1.2 times higher for individuals with no complications to almost three times higher for individuals with severe organ complications [4].

In 2011, the estimated total cost of healthcare attributed to diabetes in Denmark was €1.64 billion [4]. The steadily increasing number of individuals affected by type 2 diabetes on a global scale [5, 6] means that the extent of type 2 diabetes-related incident and prevalent cardiovascular morbidity and mortality is increasing [7]; this will eventually become of major concern to healthcare policymakers when managing limited budgets.

In the Steno-2 study, an intensified multifactorial approach was compared with a routine multifactorial intervention for 7.8 years in high-risk individuals with type 2 diabetes and microalbuminuria. Outcomes were mortality and micro- and macrovascular late complications, and the study had a total follow-up of 21.2 years. At the end of follow-up, individuals originally randomised to intensified therapy survived for a median of 7.9 years longer and incident CVD was delayed by 8.1 years with a relative risk reduction of 45% and an absolute risk reduction of 20% [8]. Further, the risk of nephropathy [9] and retinopathy [8], as well as heart failure [10], was substantially decreased in the intensified therapy group.

In a study from 2008 with modelled data from the same Steno-2 cohort, we predicted that intensified multifactorial therapy together with outpatient consultations with endocrinologists would cost more than the conventional multifactorial therapy over a period of 30 years, but would still be cost-effective because of the marked health benefits derived from intensified care [11].

In this post hoc analysis, the aim was to compare the long-term economic implications of intensified multifactorial treatment and conventional multifactorial treatment with later intensification in high-risk individuals with type 2 diabetes and microalbuminuria from the perspective of a Danish healthcare provider. Here, we have included the total realised direct medical costs for the entire 21.2 years of follow-up in the Steno-2 study.

## Methods

The study was based on the randomised Steno-2 study. Individuals were recruited from the Steno Diabetes Center, Copenhagen, Denmark, during 1993–1994. All were diagnosed with type 2 diabetes and microalbuminuria (urinary albumin excretion rate of 30–300 mg/24 h) and were thus at high risk of developing micro- and macrovascular organ damage [12]. A total of 160 individuals were randomly assigned (using sealed envelopes) to either: intensified multifactorial treatment at the Steno Diabetes Center that targeted all known modifiable risk factors, using both behavioural and pharmacological approaches; or conventional multifactorial treatment from their general practitioner (GP) following standard guidelines according to the 1988 recommendations of the Danish Medical Society, which were applicable until 2000 [12]. For an overview of risk factors treated and treatment targets in the two groups, please refer to electronic supplementary material (ESM) Table 1. After 7.8 years of intervention, all individuals were instructed in intensified multifactorial treatment and the trial continued as an observational follow-up study for an additional 13.4 years. Between 2006 and 2014, one individual in the conventional treatment group emigrated and was thus lost to follow-up (see ESM Fig. 1). A detailed description of the design of the Steno-2 study has previously been reported [12].

The protocol for the current analysis was approved by the local ethics committee (Ethics Committee, Capital Region of Denmark; protocol ID number: H-KA-99035-GS, add. 41104) and by the Danish Data Protection Agency (J. Nr 2015-41-4042) and the trial was registered (ClinicalTrials.gov registration no. NCT00320008). Danish legislation does not require specific participant consent to registry-based studies, but all participants provided written informed consent at study visits.

### Outcomes

The primary outcome of the study was the difference in total direct medical costs for the entire study duration (1993–2014) between the original randomised groups (Table 1). The secondary outcomes were the difference in costs per person-year (Table 1), and the difference in costs attributable to specific services outlined below (see Table 2). As a confirmatory analysis, we performed similar evaluations for the period 1996–2014 because of the quality of data (as described in a subsequent section).

**Table 1:** Total direct healthcare costs in the two treatment groups

Costs	Sum of healthcare costs (€)		*p* value
	Intensive	Conventional	
All direct medical			
1993–2014	12,126,900	11,181,700	0.48
1996–2014	9,850,964	9,304,795	0.64
Per person-year			
1993–2014	8996	9723	0.23
1996–2014	8725	10,091	0.045

Data from 1993 to 1996 were assessed separately because of missing data

**Table 2:** Direct costs of specific healthcare services per individual per year in the two treatment groups

Costs	Mean yearly healthcare costs per individual (€)		*p* value
	Intensive	Conventional	
Outpatient services (from 1996)			
CVD	92	136	0.087
Not CVD	1177	1211	0.99
Total	1269	1347	0.92
Inpatient admissions services			
1993–1995			
CVD	1290	2015	0.72
Not CVD	3712	2361	0.29
Total	5003	4375	0.89
From 1996			
CVD	1338	2262	0.0048
Not CVD	2957	3509	0.43
Total	4295	5771	0.028
1993–2014			
CVD	1331	2213	0.0024
Not CVD	3079	3281	0.63
Total	4410	5494	0.042
Prescription drugs			
1993–1995	5071	3410	<0.0001
From 1996	2680	2387	0.0006
Primary healthcare sector (from 1993)	433	514	<0.0001

As not all data from 1993 to 1995 are available, the periods before and after 1996 are reported separately. ‘Outpatient services’ includes any expenses related to consultations and treatment at a hospital that are not an admission. ‘Inpatient admissions services’ includes any expenses related to hospital admissions. ‘Prescription drugs’ includes any expenses related to prescribed medication. ‘Primary healthcare sector’ includes any expenses related to consultations with, or treatments from, a GP or specialised medical professional not employed at a hospital, such as an ophthalmologist

Total direct costs were divided into four categories. ‘Outpatient services’ covers any expenses attributable to consultations and treatments at a hospital that are not counted as an admission. ‘Inpatient admission services’ covers any expenses attributable to hospital admissions. ‘Prescription drugs’ covers expenses related to prescribed drugs. ‘Primary health sector’ covers costs attributable to healthcare in general practice and specialised medical professionals not employed at a hospital, such as ophthalmologists, physiotherapists and chiropractors.

Additionally, outpatient and inpatient services were split into two groups: expenses related to CVD and expenses not related to CVD (see Table 2). CVD was defined in accordance with a previous Steno-2 study publication [8].

### Data sources and definitions

Data on costs were gathered from Danish health registries and were calculated as total direct medical costs, costs per person-year as well as mean annual expenses. The National Patient Registry contains data on inpatient and outpatient services, which can be linked to the Danish Case Mix System (Diagnosis-Related Groups, DRG), thereby allowing the associated costs to be calculated [13, 14]. Expenses were retrieved for the periods 1993–2014 and 1996–2014, depending on availability of data. As data on inpatient expenses were only available from 1996, costs of inpatient services during 1993–1995 were imputed to the entire study period dataset using data on number of admission days in 1993–1995 multiplied by the mean price per day of admission. The calculated mean costs are based on the costs per day of admission during 1996–1998 and calculated for each chapter of the International Statistical Classification of Diseases and Related Health Problems, 10th revision (ICD-10; http://apps.who.int/classifications/icd10/browse/2016/en).

Data on expenses for outpatient visits and prescription drugs were not available before 1996. Drug expenses from 1996 were calculated by multiplying the retail price with the prescribed quantity. These data are available from the Danish Medicines Agency. As data were not available before 1996, it was not possible to include the observed values for outpatient costs and medical expenses for the period 1993–1995, and therefore we performed a secondary analysis covering the period 1996–2014 in addition to the primary analysis covering the full follow-up period. To mitigate the potential bias from unavailable data on prescription medicines for the period 1993–1995, we imputed data on expenses for prescription medicines in 1996 (stratified for treatment randomisation group, age group and sex) to the period 1993–1995. Because of the stepwise introduction of medicines during the period from baseline to 1995, we anticipate that this approach overestimates the real costs associated with prescription medicines.

Data on primary healthcare expenses are available from 1993 and were retrieved from the Danish National Health Insurance Service Registry. All expenses were retrieved prospectively from the index date, i.e. year 1 was defined as the first 12 months after index date—the date of the specific individual’s enrolment in the study—year 2 as the next 12 months, and so forth. The analysis followed the index year, not calendar year, as the index year was either 1993 or 1994. This means that observation period 1 was not in the same calendar year for all individuals.

To mitigate missing data on outpatient services and medication from before 1996, numbers from an overview of medication use and consultations in the two treatment groups in 1993–1995 (Table 3) were used as indicators of the differences between the two groups when registry data were not available.

**Table 3:** Courses, consultations and medication during the years 1993–1995

Intervention	Intensive group (*n* = 80)	Conventional group (*n* = 80)	Difference
Courses and training^a^			
Dietary guidance (group sessions) (h)	32	0	32
Dietary guidance (individual session) (h)	2	1	1
Smoking cessation course (group session) (h)	15	0	15
Medical consultations^b^			
Number of yearly medical consultations	5	4^c^	1^c^
Medication^d^			
Sulfonylurea, *n* (%)	40 (51)	38 (48)	2
Metformin, *n* (%)	41 (53)	17 (21)	24
Insulin, *n* (%)	25 (32)	20 (25)	5
Statin, *n* (%)	4 (5)	1 (1)	3
ACE inhibitor, *n* (%)	76 (97)	19 (24)	57
Thiazide diuretic, *n* (%)	29 (37)	4 (5)	25
Loop diuretic, *n* (%)	11 (14)	12 (15)	−1
Calcium antagonist, *n* (%)	19 (24)	9 (11)	10
Beta-blocker, *n* (%)	3 (4)	0 (0)	3

^a^Total number of education hours spent in the treatment groups during the period from baseline (1993) until the end of 1995

^b^Yearly number of medical consultations from baseline until the end of 1995 in the two treatment groups. All individuals in the conventional treatment group had consultations with their GP, except for 12 individuals receiving insulin treatment at the time of inclusion and 20 individuals at the end of 1995. The frequency of visits to GPs was assessed by questionnaire

^c^Individuals in the conventional treatment group had consultations in primary care and not in outpatient clinics (unless they were insulin treated), as in the intensified treatment group

^d^Number of individuals in each treatment group treated with different groups of medications. Data comprise self-reported drug intake assessed at the study follow-up visit in 1995. All study participants provided data. At the study follow-up visit in 1995 there were 80 individuals in the conventional treatment group and 78 individuals in the intensive treatment group

All costs were converted to euros (using a conversion factor of 7.45 Danish kroner [DKK] per €) and adjusted to 2015 price levels using the consumer price index.

### Statistics

The statistical significance of the differences in costs between the two treatment groups was assessed by bootstrap *t* test analysis with 10,000 resamples. At baseline, the two treatment groups were well balanced for demographic variables and risk factor levels, and thus we did not control for confounders [8, 12].

**Fig. 1:**
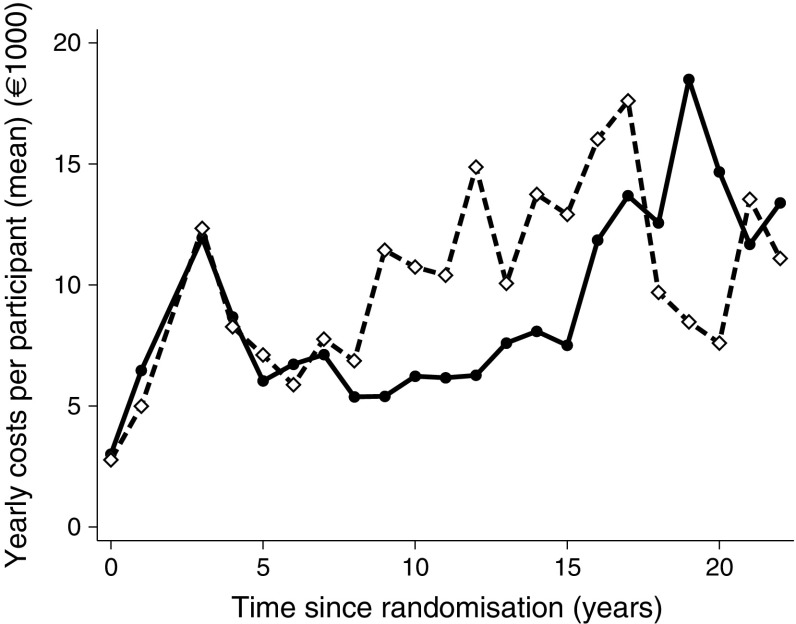
Yearly mean direct medical costs per individual (in €) in the two original treatment groups, starting at baseline in 1993. Data are the yearly costs for the group divided by the number of individuals in the group in that year. The peak at 4 years is an artefact from the aggregation of data from years 3 and 4 in some individuals. Solid line, intensified therapy group; dashed line, conventional therapy group

**Fig. 2:**
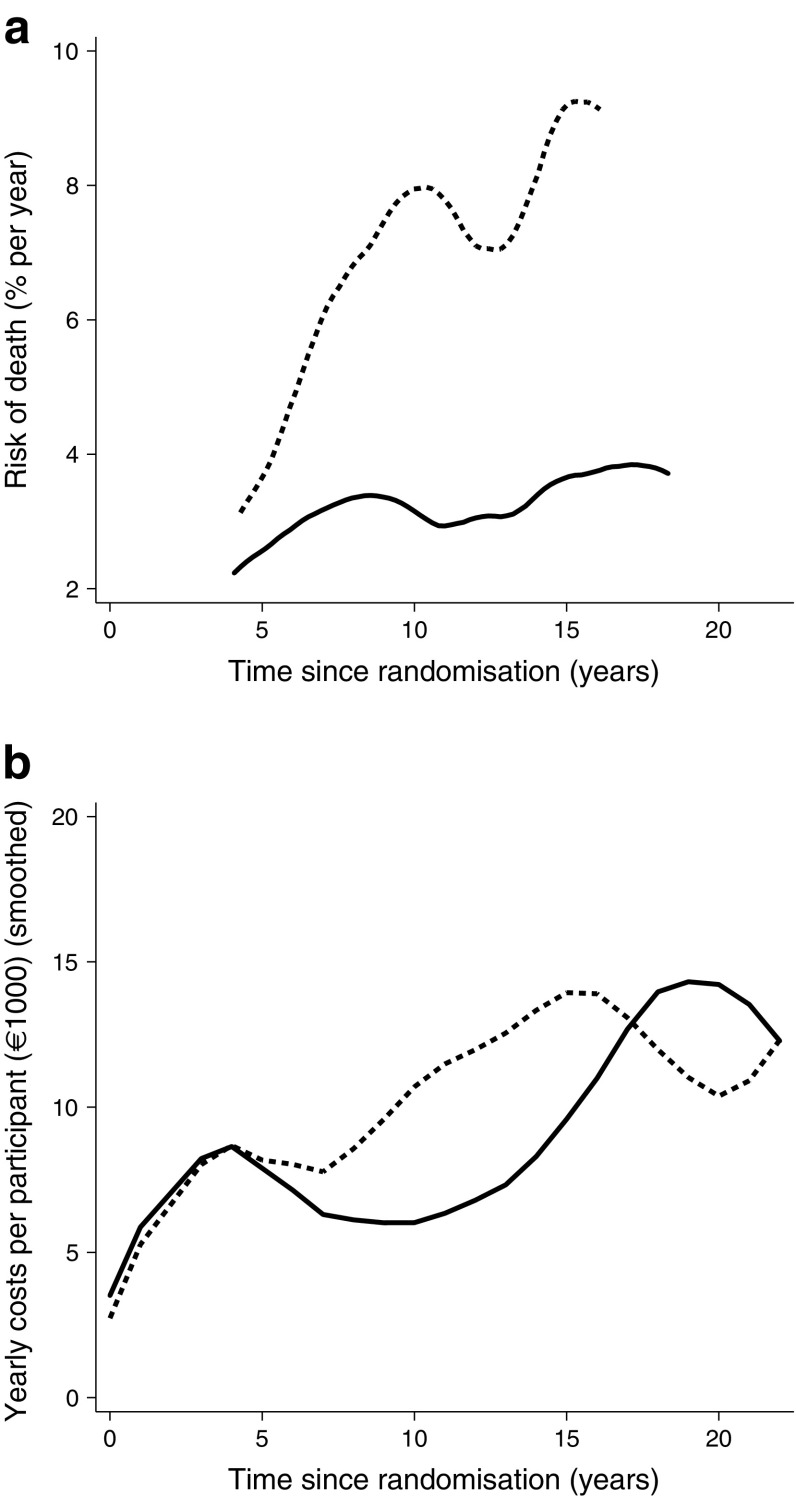
(**a**) Risk of death in the intensified therapy group (solid line) and in the conventional therapy group (dashed line). The graphs are plotted to start after baseline and are truncated before the end of follow-up because no deaths occurred in those periods, hence the hazard was not calculated. (**b**) Yearly direct health costs (as in Fig. 1), with data smoothed. Comparing the two graphs shows similar trends (with allowance for minor delay in the cost curve because of the way data were collected). Increasing mortality is followed closely by increased expenditure, reflecting increased morbidity prior to death

In Figs 1 and 2 there appears to be a peak in costs during year 4, but this is an artefact stemming from the aggregation of data from years 3 and 4 in some individuals. Smoothed mean cost curves were constructed using locally weighted scatterplot smoothing (LOWESS) with a bandwidth of 0.4.

In Fig. 2a, the hazard of death was calculated using a weighted kernel-density estimate with a default bandwidth, i.e. as a rolling mean of the hazard in the adjacent time period. Figure 4 was constructed using all cumulative cardiovascular events, amputations, end-stage renal disease and blindness events per individual included (i.e. multiple events per individual allowed).

The data used for these analyses were those collected and previously reported for the long-term follow-up of clinical outcomes and survival [8]. Statistical analyses were performed blinded for treatment allocation. SAS version 9.4 was used for the statistical analyses, while figures were constructed using STATA version 15 (STATA, College Station, TX, USA).

## Results

Each group comprised 80 randomised individuals. Selected baseline characteristics are presented in Table 4. The intensified treatment group was observed for a total of 1310 person-years and the conventional therapy group for 1108 person-years.

**Table 4:** Baseline demographic data

Characteristic	Baseline demographics (1993)	
	Intensive (*n* = 80)	Conventional (*n* = 80)
Age, years, mean ± SD	54.9 ± 7.2	55.2 ± 7.2
Age range, years	37–67	42–67
Proportion male sex, %	79	70
Known diabetes duration, years, median (range)	4 (0–30)	6 (0–29)

We found no statistically significant difference in total direct medical costs between the two groups for the entire follow-up period (1993–2014). The total costs in the intensified treatment group were €12,126,900 compared with €11,181,700 in the conventional treatment group (*p* = 0.48) (see Table 1). In the same period, the cost per person-year was €8996 in the intensified treatment group and €9723 in the conventional treatment group (*p* = 0.23) (see Table 1).

For the partial follow-up period (1996–2014), no significant difference in total costs was found between the two groups. The total costs were €9,850,964 in the intensified treatment group and €9,304,795 in the conventional treatment group (*p* = 0.64). The mean cost per person-year was lower in the intensified therapy group for the period 1996–2014, with an expense per person-year of €8725 in the intensified treatment group and €10,091 in the conventional treatment group (*p* = 0.045).

Table 2 shows an overview of the mean annual expenses per person in the intensified and conventional treatment groups, respectively, in the different categories. In the intensified therapy group, yearly expenses for prescription drugs were higher than in the conventional therapy group. Using the imputed data from 1993–1995, we found that the average yearly costs of prescription drugs during this period 1993–1995 were higher in the intensified treatment group compared with the conventional treatment group (*p* < 0.0001). The same pattern was seen during the remaining 18 years of follow-up, *p* = 0.0006. In contrast, yearly expenses for primary care and inpatient admissions were significantly lower in the intensified therapy group compared with the conventional therapy group (*p* < 0.0001 and *p* = 0.042, respectively). The difference in yearly inpatient health costs was driven by increased costs for CVD-related admissions in the conventional treatment group (*p* = 0.0024). We found no significant differences in yearly expenses for outpatient costs per person (*p* = 0.92).

**Fig. 3:**
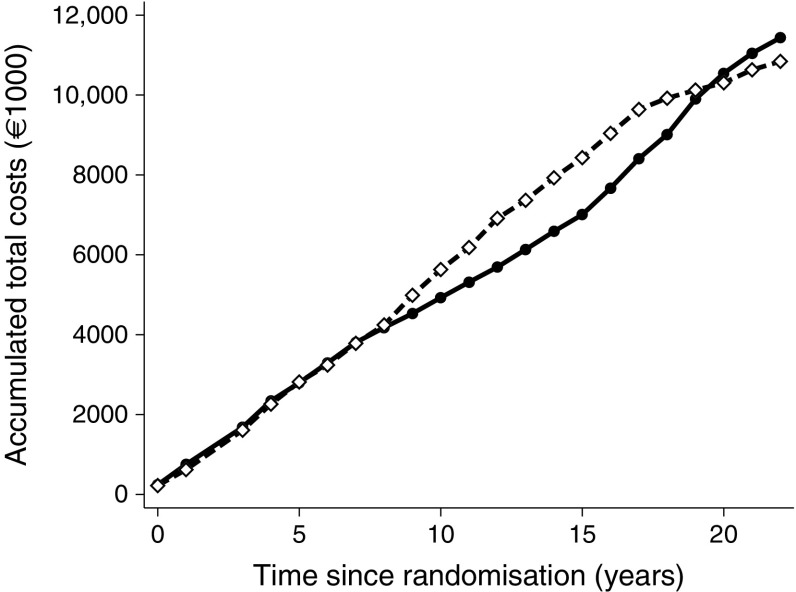
Total direct medical costs for all individuals in each treatment group. Solid line, intensified therapy group; dashed line, conventional therapy group

When observing the annual costs per individual (Fig. 1), the two treatment groups have similar expenditures during the first 8 years. After 8 years, the yearly costs per individual rise steeply in the conventional treatment group, but stay essentially unchanged in the intensified treatment group. After 15 years, however, the yearly costs in the intensified treatment group start to increase, resembling the increasing costs of the conventional treatment at year 8. Figure 2 shows yearly costs and mortality hazard curves for people in the two treatment arms compared; it appears that increased costs follow mortality rates as a proxy of higher level of morbidity (prior to death). These findings are mirrored in the cumulative costs (Fig. 3). This pattern reflects our previous finding that CVD and mortality were delayed by about 8 years in the intensified therapy group [8]. After 17 years, however, the slope of the conventional treatment group starts to flatten, corresponding with the declining yearly costs seen in Fig. 1, and the cumulated costs of the two groups start converging, meeting at year 19. Hereafter, the intensified treatment group has the highest cumulative costs, reflecting the higher number of persons alive at the end of the follow-up period in the intensified treatment group (*n* = 42 in the intensified treatment group and *n* = 24 in the conventional treatment group).

## Discussion

The overall conclusion of the 21.2 years of follow-up in the Steno-2 study was that intensified multifactorial intervention increased lifespan by a median of 7.9 years and similarly postponed incident CVD by 8.1 years [8]. In the present post hoc analysis of the total direct medical costs in the Steno-2 study, we show that intensified multifactorial intervention in individuals with type 2 diabetes and microalbuminuria is cost-neutral compared with conventional multifactorial intervention over 21.2 years of follow-up. Furthermore, we demonstrate that the cost per person-year was lower in the intensified therapy group compared with the conventional treatment group in the period 1996–2014, which is the period where complete data were available. We found that the main driver of the reduced cost per person-year in the intensified therapy group was inpatient care consequent to CVD admission because the total number of events in the conventional treatment group was double that in the intensified treatment group [8].

These results support our hypothesis that the increased costs of medication and more frequent outpatient contacts early in the disease course associated with a more intensive treatment regimen were offset by the lower risk of late complications and associated health costs.

Based on the non-significant difference in total costs between groups for the full follow-up of approximately €1,000,000 for the 202 more person-years in the intensive therapy group during follow-up, a rough estimate of €5000 per life-year gained could be calculated. Despite this calculation not being performed in a formal manner because of data limitations and not being corrected for quality of life (which would yield a cost per quality-adjusted life-year [QALY] outcome), this figure suggests that intensified multifaceted intervention in high-risk individuals with type 2 diabetes is highly feasible when balancing healthcare costs and treatment benefits in a Danish healthcare setting.

As the Steno-2 study included high-risk individuals with type 2 diabetes and microalbuminuria, findings from this analysis may not be generalisable. However, recently published real-world evidence demonstrates an improved prognosis of individuals with type 2 diabetes over recent decades, reflecting the widespread implementation of intensified multifactorial approaches to management of type 2 diabetes in general [7, 15].

After 8 years, we observed an acceleration in costs in the conventional therapy group. This probably reflects individuals in the conventional treatment group accruing more non-fatal events, adding to their overall morbidity and resulting in an increased need for and use of medication, as well as more frequent outpatient visits (Fig. 2). The intensification of the diabetes treatment regimen after 7.8 years may also have contributed to increased costs.

**Fig. 4:**
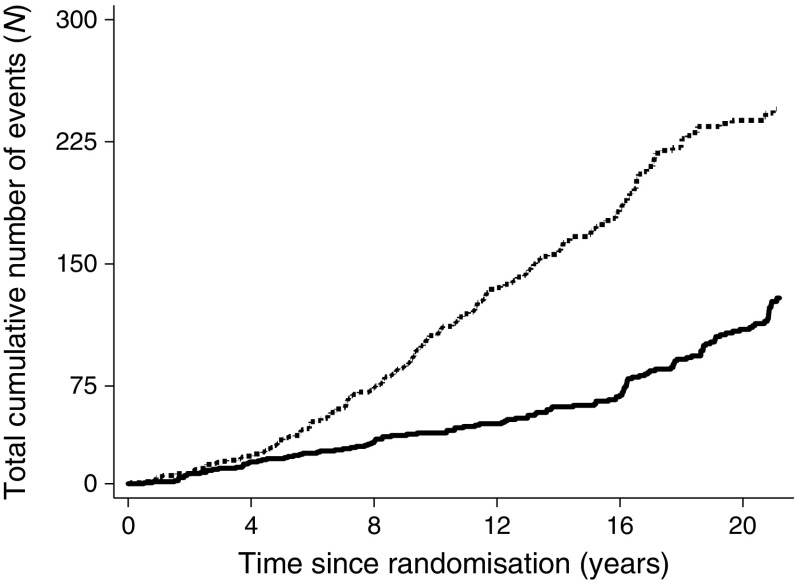
Cumulative number of events including myocardial infarction, stroke, amputation, revascularisation, blindness, end-stage renal disease and death. Recurrent events were included. Solid line, intensified therapy group; dashed line, conventional therapy group

After about 17 years, there was a steep decline in the yearly expenses per individual in the conventional treatment group. During the later years, however, the sample sizes of the two treatment arms diminished and, as a result of this, a single individual had a higher influence on the overall results. Therefore, when analysing and interpreting the fluctuations in expenses towards the end of the study period, much caution should be taken, as the death of a single, yet health-cost-demanding individual (e.g. one in chronic renal replacement therapy) may have had a considerable impact on the total health expenses.

Expenses for individuals in chronic dialysis treatment have been imputed in the overall analyses, but the requirement for anonymity meant it was not possible to analyse this specific complication separately.

In the analyses, we used high-quality Danish registries containing complete information covering all individuals in the majority of the study period, the direct medical costs from the primary and secondary health sector and the use of drugs. However, the costs of prescribed medication and outpatient services were unavailable for the first 3 years after randomisation. The costs of inpatient admission services from 1993 to 1995 were estimated based on the number of admission days and the mean daily costs for an admission in a certain ICD10 grouping. However, the majority of complications occurred after the first 3 years of intervention, as shown in Fig. 4, which means the potential for bias from incomplete data is low.

At the end of the first 3 years, the largest differences in use of medication between the intensified treatment group and the conventional treatment group were seen for metformin (difference = 24 individuals), ACE inhibitors (difference = 57 individuals), thiazide diuretics (difference = 25 individuals) and, to a lesser extent, calcium antagonists (difference = 10 individuals). All these drugs were non-generic in the majority of the formal intervention period. For medical consultations during 1993–1995, the consultations in the conventional treatment group were mainly done in the primary sector and are thus already accounted for in all analyses. However, individuals in the conventional treatment group who were using insulin treatment were seen in outpatient clinics on average five times yearly, as in the intensified treatment group (*n* = 20 at the end of 1995). Therefore, the consultations of the 20 individuals in the conventional treatment group along with the consultations in the intensified treatment group are not accounted for in the primary analysis during this period.

In 2008, we reported a cost-effectiveness analysis using modelled data in the same Steno-2 cohort based on data from the initial 13 years of observation and projecting clinical and cost outcomes over participant lifetimes [11]. It was anticipated that intensified multifactorial treatment in an outpatient setting would be more expensive than conventional treatment over a period of 30 years, primarily because of increased medication costs and specialised treatment. This is somewhat in line with the actual results of the current study, showing that the intensified treatment was associated with higher medication expenses and slightly more consultations.

In the above-mentioned cost-effectiveness analysis, an incremental cost-effectiveness ratio (ICER) of €2538 per QALY was calculated, which was considered cost-effective [11]. Because a direct QALY measure was not included in the Steno-2 study, the two sets of analysis outcomes are not directly comparable. However, in the present study, the increased lifespan and cost-neutrality align well with the predicted low cost per QALY gained.

In our study, we were not able to collect data on productivity, tertiary care or rehabilitation, which presumably accounted for substantial costs. The total costs of tertiary care may increase following intensified treatment, as individuals live longer. Conversely, the reduced incidence of strokes, amputations, end-stage renal disease, blindness and other disabling organ complications in the intensified treatment group might result in a longer productive period as part of the work force [16]. Similarly, a reduction in the need for rehabilitation and tertiary care in the originally intensively treated individuals may point to a smaller financial burden for society.

The emergence of generic drugs for treating all the major risk factors (hyperglycaemia, hypertension, dyslipidaemia and increased platelet aggregation and hypercoagulation) will allow most individuals with type 2 diabetes to benefit from an intervention regimen similar to the intensified therapy group, with costs of pharmaceutical drugs vastly reduced compared with the original 7.8 years of intensified intervention in the Steno-2 study. This therapeutic approach may allow for improvements in the prognosis of people with type 2 diabetes in low-income settings.

Since the completion of the Steno-2 study, guidelines for the treatment of type 2 diabetes have emphasised the importance of multiple risk factor intervention [17]. Consequently, the risk for major CVD events in this population has decreased [7]. However, it is evident from recent large clinical trials that a substantial proportion of individuals are not treated according to guidelines. In the LEADER (Liraglutide Effect and Action in Diabetes: Evaluation of Cardiovascular Outcome Results) and EMPA-REG OUTCOME trials, despite enrolled individuals being at very high risk of micro- and macrovascular complications, around 25% of participants were not treated with statins, around 20% were not receiving renin–angiotensin–aldosterone (RAAS) inhibitors and 10–20% were not receiving anticoagulants, in contrast with guideline recommendations [18–21]. In addition, newer drugs, such as glucagon-like peptide 1 (GLP-1) analogues and sodium/glucose cotransporter 2 (SGLT-2) inhibitors, have a special role in the treatment algorithm of high-risk patients with type 2 diabetes [22]. Therefore, we believe that the comparison between intensified therapy and less-intensive therapy is still relevant today.

Nevertheless, the overall results from the present analysis from the Steno-2 study demonstrate that increased use of economic resources spent on an early multipronged intensive treatment strategy (e.g. on medication and specialist consultations) prevents diabetes-related complications, whereas conventional treatment requires spending the same amount, but on treating quality- and length-of-life-reducing complications. With continuous drug development and consequently new drugs available to further reduce risk, drugs will likely continue to be an important expense in future diabetes care. Still, diabetes complications are the main driver of direct medical costs in type 2 diabetes [4] and therefore it is important for decision-makers to incorporate the assessment of clinical outcomes of importance, and not just direct costs and surrogate outcome markers, in the evaluation of new treatment opportunities.

In conclusion, over 21.2 years of follow-up, the total direct costs of an intensified multifactorial intervention, which led to a disease-free life-length improvement of about 8 years, were similar to those of conventional treatment. The major limitation of the study was missing data on expenses related to prescription medicines and inpatient admissions during the first 3 years of the trial, the missing data on tertiary care and rehabilitation and the small study sample size. However, our data clearly suggest that investing in early intensified intervention in high-risk individuals with type 2 diabetes in a Danish healthcare setting will pay for itself over time through reductions in the cost of treating diabetes complications.

## Electronic supplementary material

ESM (PDF 490 kb)

## Data Availability

The data are held on a secure server at Statistics Denmark and are accessible only to individuals with special authorisation for the project. Aggregate-level data are available at reasonable request to the corresponding authors.

## Abbreviations

CVD: Cardiovascular disease
GP: General practitioner
QALY: Quality-adjusted life-year
