# Supplementary material for: A cost analysis of intensified vs conventional multifactorial therapy in individuals with type 2 diabetes: a post hoc analysis of the Steno-2 study
Source: Diabetologia. 2018 Oct 6;62(1):147–55. doi: 10.1007/s00125-018-4739-3 (PMC6290682; doi:10.1007/s00125-018-4739-3)
Supplement: Supplementary file 1 — (PDF 490 kb) [file 125_2018_4739_MOESM1_ESM.pdf]

Electronic supplementary materials

ESM Table 1: Treatment targets

	Intensive		Conventional	
	1993-1999	2000-2001	1993-1999	2000-2001
Systolic blood pressure (mm Hg)	< 140	< 130	< 160	< 135
Diastolic blood pressure (mm Hg)	< 85	< 80	< 95	< 85
HbA_{1c} (%)	< 6.5	< 6.5	< 7.5	< 6.5
HbA_{1c} (mmol/mol)	< 48	< 48	< 58	< 48
Fasting serum total cholesterol (mmol/l)	< 4.9	< 4.5	< 6.5	< 4.9
Fasting serum triacylglycerol (mmol/l)	< 1.7	< 1.7	< 2.2	< 2.0
Treatment with ACE-inhibitor irrespective of BP	Yes	Yes	No	Yes
Aspirin therapy				
Known ischemia	Yes	Yes	Yes	Yes
Peripheral vascular disease	Yes	Yes	No	No
No known vascular disease	No	Yes	No	No

Treatment targets for treatments group during the initial 7.8 years of the Steno-2 trial. The conventional therapy group were at all times treated with targets as least as strict as recommended in national guidelines. Any behavioural and pharmacological intervention was allowed in order to achieve targets. Aspirin treatment was initiated if any of the indication criteria were met. The mean intervention duration was 7.8 years and hereafter all patients were offered treatment similar to that of the original intensive therapy group.

ESM Fig. 1

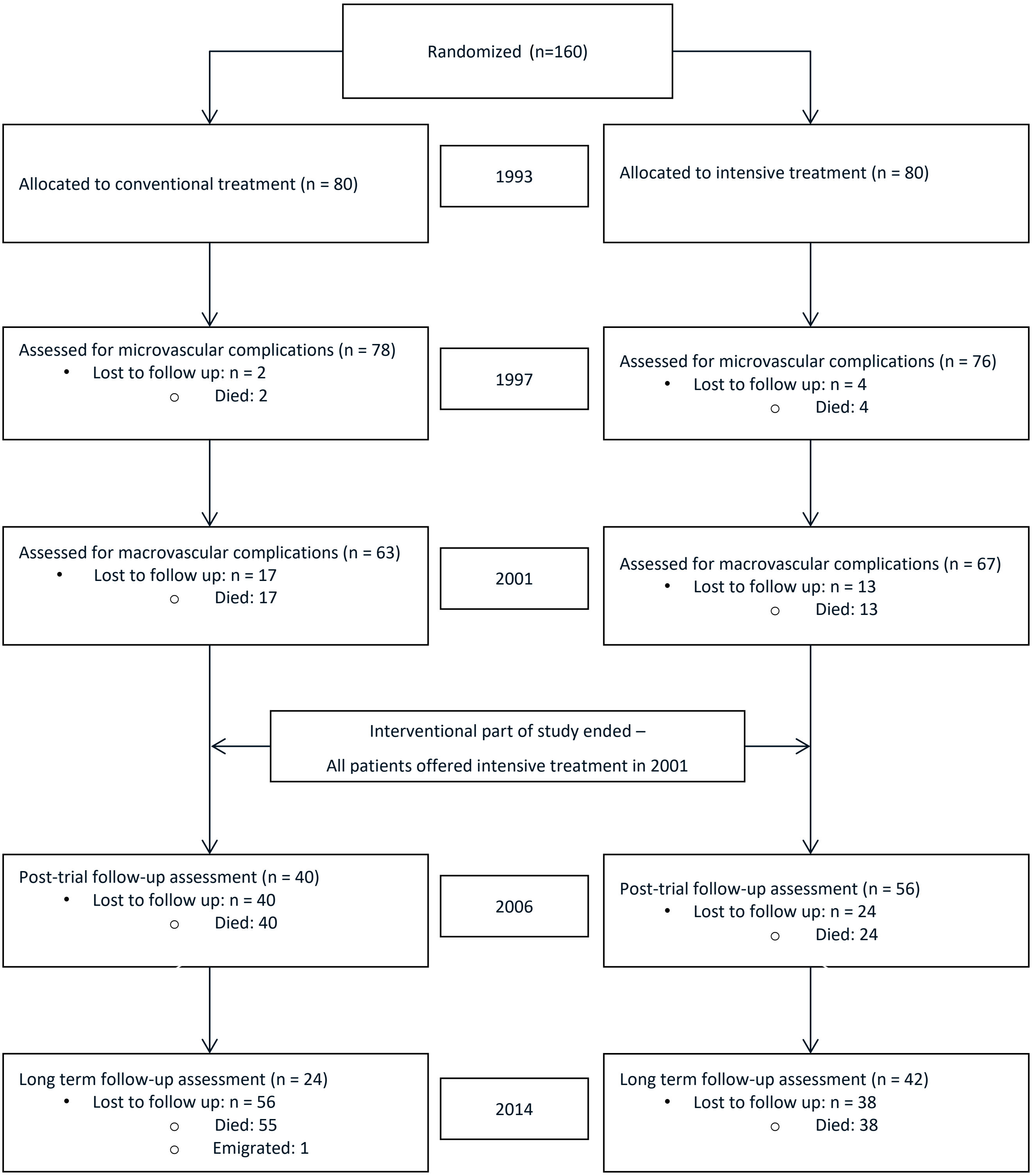

ESM Fig. 1: Consort diagram showing the flow of patients in the two treatment groups of the Steno-2 trial and post-trial follow-up. The intervention was neutralised after a mean of 7.8 years and the study continued as an observational follow-up trial with all patients being suggested intensified treatment.
